# Past alcohol consumption and incident atrial fibrillation: The Atherosclerosis Risk in Communities (ARIC) Study

**DOI:** 10.1371/journal.pone.0185228

**Published:** 2017-10-18

**Authors:** Shalini Dixit, Alvaro Alonso, Eric Vittinghoff, Elsayed Soliman, Lin Y. Chen, Gregory M. Marcus

**Affiliations:** 1 Cardiac Electrophysiology Section, Division of Cardiology, Department of Medicine, University of California, San Francisco, San Francisco, CA, United States of America; 2 Division of Epidemiology and Community Health, School of Public Health, University of Minnesota, Minneapolis, MN, United States of America; 3 Department of Epidemiology and Biostatistics, University of California, San Francisco, San Francisco, CA, United States of America; 4 Department of Epidemiology, Division of Public Health Sciences, Wake Forest University School of Medicine, Winston-Salem, NC, United States of America; 5 Cardiovascular Division, Department of Medicine, University of Minnesota Medical School, Minneapolis, MN, United States of America; Medizinische Fakultat der RWTH Aachen, GERMANY

## Abstract

**Background:**

Although current alcohol consumption is a risk factor for incident atrial fibrillation (AF), the more clinically relevant question may be whether alcohol cessation is associated with a reduced risk.

**Methods and results:**

We studied participants enrolled in the Atherosclerosis Risk in Communities Study (ARIC) between 1987 and 1989 without prevalent AF. Past and current alcohol consumption were ascertained at baseline and at 3 subsequent visits. Incident AF was ascertained via study ECGs, hospital discharge ICD-9 codes, and death certificates. Of 15,222 participants, 2,886 (19.0%) were former drinkers. During a median follow-up of 19.7 years, there were 1,631 cases of incident AF, 370 occurring in former consumers. Former drinkers had a higher rate of AF compared to lifetime abstainers and current drinkers. After adjustment for potential confounders, every decade abstinent from alcohol was associated with an approximate 20% (95% CI 11–28%) lower rate of incident AF; every additional decade of past alcohol consumption was associated with a 13% (95% CI 3–25%) higher rate of AF; and every additional drink per day during former drinking was associated with a 4% (95% CI 0–8%) higher rate of AF.

**Conclusions:**

Among former drinkers, the number of years of drinking and the amount of alcohol consumed may each confer an increased risk of AF. Given that a longer duration of abstinence was associated with a decreased risk of AF, earlier modification of alcohol use may have a greater influence on AF prevention.

## Introduction

Atrial fibrillation (AF) affects millions of Americans and is growing in incidence and prevalence [1]. It is one of the most common causes of stroke and nearly doubles mortality [2, 3]. While many efforts have concentrated on the treatment of this important disease, primary prevention has rarely been a focus [4].

Several studies have found an association between heavy alcohol consumption, usually defined as greater than 3 drinks per day, and incident AF [5–7]. Though the data on moderate alcohol consumption is less conclusive, a meta-analysis of fourteen studies determined that there is a linear relationship between alcohol consumption and AF, reporting an 8% increase in risk associated with each 10 grams per day of alcohol intake [8]. A study from our group demonstrated that even greater access to alcohol is associated with a higher prevalence and incidence of AF [9]. In addition, we recently identified alcohol consumption as a predictor of left atrial enlargement and subsequent incident AF, elucidating a possible mechanism for this relationship [10]. Thus, eliminating alcohol intake could be a potentially effective strategy for preventing AF. As of now, it remains unknown whether once an individual has consumed alcohol, the “genie is out of the bottle” or whether cessation may still influence AF risk.

## Methods

### Study population

The ARIC study is a population-based prospective cohort designed to identify risk factors for atherosclerosis and cardiovascular disease. Between 1987 and 1989, 15,792 adults aged 45 to 64 were recruited from 4 U.S. communities: Forsyth County, NC; Jackson, MS; Minneapolis suburbs, MN; and Washington County, MD [11]. After a comprehensive baseline assessment, participants underwent four follow-up visits in 1990–92, 1993–95, 1996–98, and 2011–13 and were contacted yearly by phone to obtain information about hospital admissions and to ascertain vital status. For this analysis, we first excluded individuals with missing alcohol information (n = 324), then those without an ECG examination (n = 214), and then those with prevalent AF (n = 32), for a total of 15,222 participants. Prevalent AF was defined as AF present at baseline examination; these participants were excluded in order to examine specifically new-onset AF. The ARIC study was approved by the institutional review boards at the University of Minnesota, Johns Hopkins University, Wake Forest University, University of North Carolina, University of Texas Health Sciences Center at Houston, and the University of Mississippi Medical Center. All participants provided written informed consent. For this particular analysis, a certificate of approval to conduct research on this de-identified dataset was obtained from the University of California, San Francisco Committee on Human Research.

### AF ascertainment

Prevalent AF was identified by baseline ECG. Incident AF diagnoses were obtained from subsequent study visit ECGs, hospital discharge records, and death certificates [12]. Study participants underwent standard supine 12-lead resting ECG recordings at baseline and at each follow-up exam. All ECG recordings were done with MAC PC Personal Cardiographs (Marquette Electronics, Inc., Milwaukee, WI). ECGs were transmitted by telephone to the ARIC Central ECG Reading Center for coding, interpretation and storage. All ECG recordings automatically coded as AF were visually re-checked by a trained cardiologist to confirm the diagnosis [13].

Annual follow-up phone calls to the study participants were used to identify hospitalizations and deaths, and discharge lists from local hospitals were reviewed for any cardiovascular events. Using International Classification of Diseases, ninth revision, clinical modification (ICD-9- CM) codes, AF was considered present if 427.31 or 427.32 were present in a hospitalization, so long as they were not accompanied by a procedure code for open cardiac surgery. The diagnosis of AF based on hospital discharge records has been previously validated in ARIC [12]. Finally, AF was considered present if the death certificate included ICD-9 code 427.3 or ICD-10 code I48.

### Alcohol assessment

Self-reported alcohol consumption was ascertained at baseline and each subsequent visit through an interviewer-administered dietary questionnaire. Participants were asked whether they currently consumed alcoholic beverages, and, if not, whether they had in the past. Follow-up questions for those citing past drinking included 1) number of years since the participant stopped drinking, 2) number of years the participant drank for, 3) types of alcoholic beverages consumed in the past, and 4) usual number of drinks per week before stopping. The amount of alcohol consumed currently and in the past in grams per week was then calculated assuming the following alcohol content: 4 oz of wine, 10.8 g; 12 oz of beer, 13.2 g; and 1.5 oz of hard liquor, 15.1 g [14, 15].

### Additional covariate ascertainment

At baseline, sex, race, education level, and smoking history were obtained from self-report. Level of education was divided into 3 categories: some high school or less, high school graduate/vocational school, some college/graduate school based on previous ARIC study analyses [14, 16]. Smoking status was classified as current, former, or never smoker, and cigarette years of smoking were calculated from the average number of cigarettes smoked per day times the number of years smoked for both current and former smokers. Body mass index (BMI, kg/m^2^) was calculated from baseline height and weight measurements. Diabetes mellitus (DM) was defined as a fasting glucose ≥126 mg/dL (or non-fasting glucose of ≥200 mg/dL), a self-reported physician diagnosis of diabetes, or current use of diabetes medication. Hypertension (HTN) was defined as a blood pressure measurement of ≥140 mmHg systolic and/or ≥90 mmHg diastolic, or current use of antihypertensive medication. Prevalent coronary artery disease (CAD) was defined as a self-reported history of myocardial infarction (MI), coronary bypass or angioplasty, or MI indicated on baseline ECG. Prevalent heart failure (HF) was identified by the Gothenburg criteria or by current use of HF medication [17].

### Statistical analysis

Continuous variables with normal distribution are presented as means ± standard deviation (SD) and were compared using analysis of variance. Non-normally distributed continuous variables are presented as medians with interquartile ranges (IQRs) and were compared using the Kruskal–Wallis test. The association between categorical variables was determined using the chi-square test.

Cox proportional hazards models were used to investigate the associations between drinker status and past drinking characteristics and incident AF both before and after controlling for potential confounders. Checks for log-linearity of the alcohol consumption variables used as predictors were performed. Covariates were selected based on biological plausibility and past literature indicating associations with alcohol use and/or incident AF. We included study site, age, sex, race, BMI, smoking status, cigarette years, HTN, DM, CAD, and HF as potential confounders. The proportional hazards assumption was assessed using Kaplan–Meier versus predicted survival plots and log-minus-log survival plots.

Analyses of longitudinal alcohol consumption and incident AF were performed using alcohol intake as a time dependent covariate. Alcohol intake in grams per day was available for visits 1, 2, 3, and 5 and was modeled as linear between visits and stable subsequent to visit 5. From these models, we derived two different predictor variables: average daily intake from baseline to present (in grams per day) and cumulative alcohol exposure (estimated kilograms consumed over follow-up). Cox proportional hazard models were then used to investigate the associations between these predictors and incident AF both in unadjusted and adjusted models as described above.

Data were analyzed using Stata 13 (StataCorp, College Station, TX, USA). A two-tailed p<0.05 was considered statistically significant. A certificate of approval to conduct research on this de-identified dataset was obtained from the University of California, San Francisco Committee on Human Research.

## Results

After exclusion of those with prevalent AF (n = 32), there were 15,222 participants included in our analysis. At baseline, 3,790 (25%) reported being never drinkers, 8,546 (56%) current drinkers, and 2,886 (19%) former drinkers. Former consumers were more likely than current drinkers to be older, black, have a lower level of education, be former smokers, have smoked for longer, have drunk more alcohol per week (when drinking), have a larger BMI, and were more likely to have DM, HTN, CAD, and HF (Table 1). In comparing former drinkers to never drinkers, former consumers were more likely to be male, white, have a lower level of education, be current or former smokers, have smoked for longer, and have DM, CAD, and HF (Table 1).

**Table 1:** Baseline characteristics in the Atherosclerosis Risk in Communities (ARIC) study by drinking status.

	Never Drinkers (N = 3,790)	Current Drinkers (N = 8,546)	Former Drinkers (N = 2,886)	P Value
**Mean age ± SD, *y***	54.6 ± 5.8	53.8 ± 5.8	54.7 ± 5.7	<0.001
**Male, *n (%)***	864 (23)	4,442 (52)	1,550 (54)	<0.001
**Race, *n (%)***				
**White**	2,009 (53)	7,240 (85)	1,943 (67)	<0.001
**Black**	1,768 (47)	1,282 (15)	935 (32)	
**Other**	13 (0.3)	24 (0.3)	8 (0.3)	
**Education level, *n (%)***				
**High school or less**	1,203 (32)	1,306 (15)	1,103 (38)	<0.001
**High school graduate**	1,298 (34)	2,789 (33)	841 (29)	
**Vocational school**	254 (7)	790 (9)	249 (9)	
**College**	694 (18)	2,672 (31)	513 (18)	
**Graduate/ professional school**	333 (9)	977 (12)	175 (6)	
**Smoking status, *n (%)***				
**Never smoker**	2,601 (69)	2,853 (33)	835 (29)	<0.001
**Current smoker**	568 (15)	2,554 (30)	883 (31)	
**Former smoker**	615 (16)	3,133 (37)	1,166 (40)	
**Median cigarette years (IQR)**	0 (0–96)	230 (0–620)	280 (0–720)	<0.001
**Median drinks per week (IQR)**	0 (0–0)	2.3 (0–7.2)	4 (1–12)	<0.001
**Mean BMI + SD, *kg/m*^*2*^**	29 ± 6	27 ± 5	28 ± 6	<0.001
**Diabetes mellitus, *n (%)***	619 (17)	681 (8)	512 (18)	<0.001
**Hypertension, *n (%)***	1,624 (43)	2,553 (30)	1,115 (39)	<0.001
**Coronary artery disease, *n (%)***	117 (3)	380 (5)	244 (8)	<0.001
**Heart failure, *n (%)***	224 (6)	294 (4)	201 (7)	<0.001

BMI, body mass index; IQR, interquartile range. Values are reported as mean ± SD, median (IQR), or number (%).

**Fig 1:**
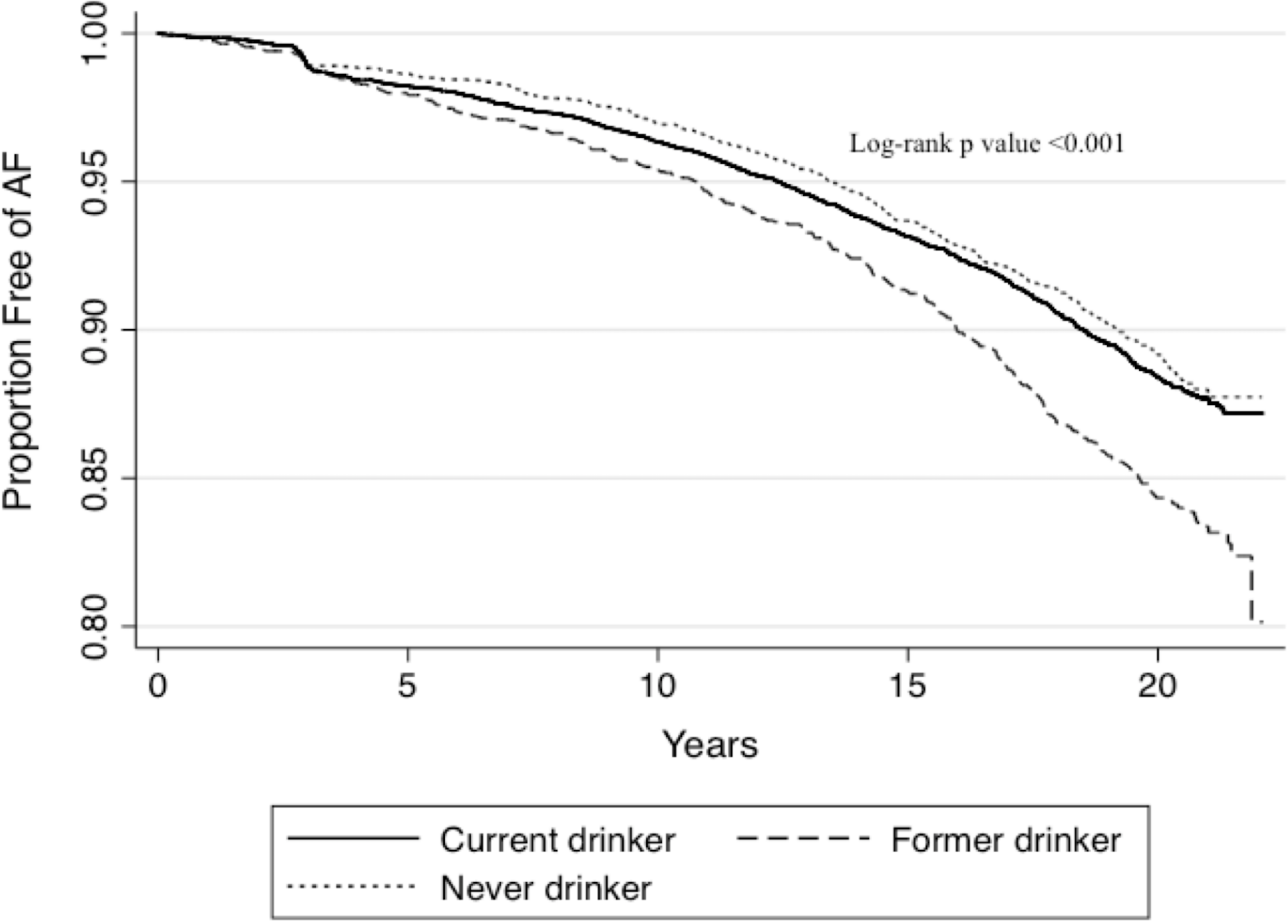
Kaplan-Meier curves for incident atrial fibrillation (AF) in Atherosclerosis Risk in Communities (ARIC) study participants by baseline drinking status. Differences in proportions with and without AF are compared using the log-rank test.

**Fig 2:**
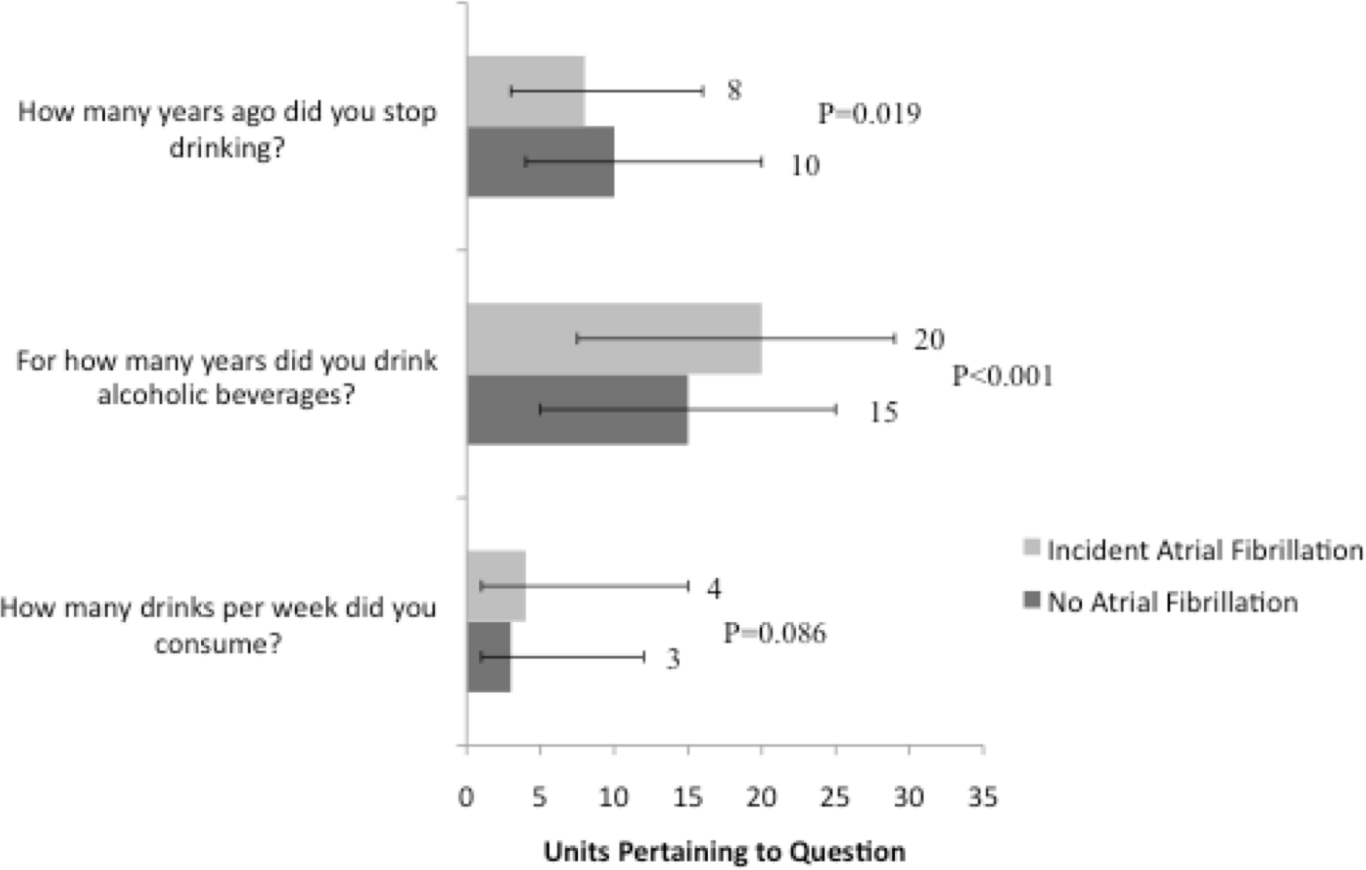
Summary of alcohol consumption patterns among former drinkers (N = 2,886) stratified by the development of incident atrial fibrillation. Values are reported as medians for numeric responses. Y error bars denote interquartile ranges.

During a median follow-up of 19.7 years, there were 1,631 cases of incident AF, 370 occurring in former consumers. Past drinkers had an increased risk of developing AF (Fig 1). Focusing on past alcohol consumers (n = 2,886), those who did not develop AF quit longer ago, drank for a shorter period of time, and consumed less alcohol in the past (Fig 2). After adjusting for study site, age, sex, race, BMI, smoking status, cigarette years, HTN, DM, CAD, and HF, every decade abstinent from alcohol was associated with an approximate 20% lower rate of incident AF; every additional decade alcohol was consumed in the past was associated with a 13% higher rate of AF; and every additional drink per day during former drinking was associated with a 4% higher rate of AF (Table 2).

**Table 2:** Risk of incident atrial fibrillation according to past alcohol consumption among former drinkers (N = 2,886).

	Unadjusted Hazard Ratio	95% Confidence Interval	P Value	Adjusted Hazard Ratio	95% Confidence Interval	P Value
**Number of Years Abstinent**	0.89*	0.81–0.99	0.033	0.80*	0.72–0.89	<0.001
**Number of Years Consumed Alcohol**	1.30*	1.19–1.42	<0.001	1.13*	1.03–1.25	0.011
**Average Number of Drinks per Day**	1.07^†^	1.03–1.10	<0.001	1.04^†^	1.00–1.08	0.040

*Hazard ratio per decade.

^†^Hazard ratio per daily drink.

Multivariate analyses were adjusted for study site, age, sex, race, body mass index, smoking status, cigarette years, hypertension, diabetes mellitus, coronary artery disease, and heart failure.

**Fig 3:**
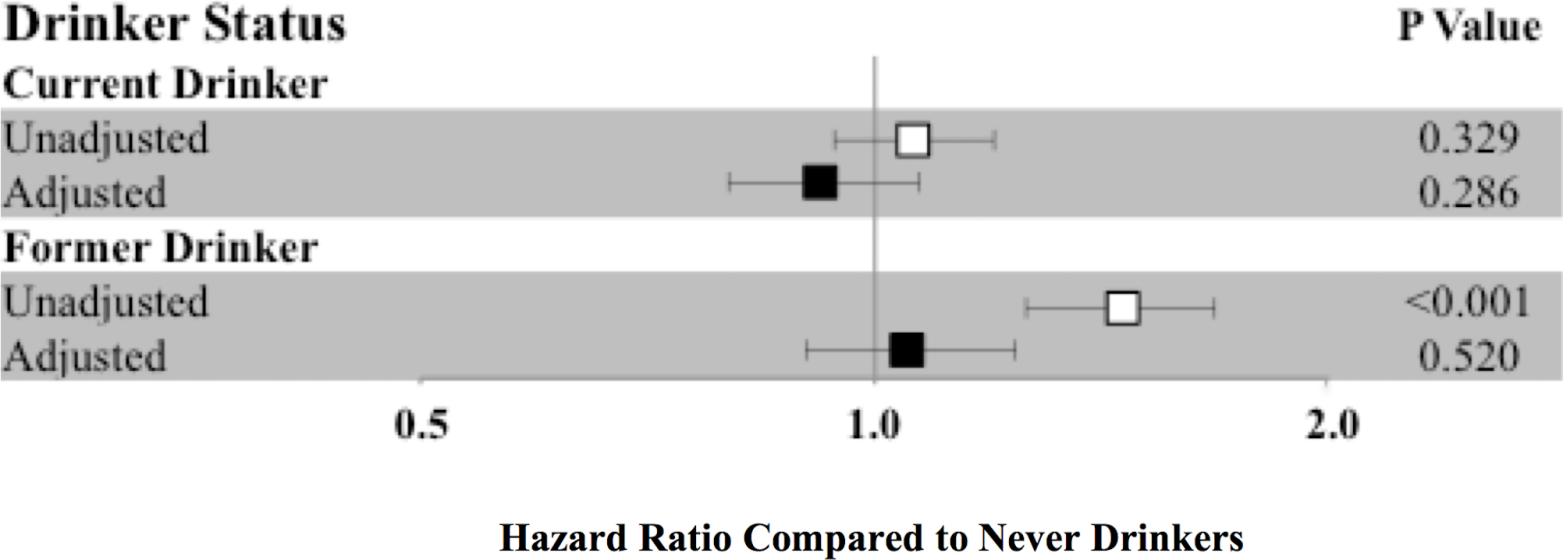
Hazard ratios for incident atrial fibrillation according to drinker status with never drinkers as the reference group. Hazard ratios are shown unadjusted (white square) and adjusted (black square) for study site, age, sex, race, body mass index, smoking status, cigarette years, hypertension, diabetes mellitus, coronary artery disease, and heart failure. Y error bars denote 95% confidence intervals.

In unadjusted analyses, former consumers had an increased risk of developing AF compared to never drinkers (HR 1.46, 95% CI 1.26–1.68, p <0.001), but, after adjusting for study site, age, sex, race, smoking status, years of smoking, BMI, HTN, DM, CAD, and HF, this relationship lost statistical significance (Fig 3). Former drinkers remained at higher risk of AF when compared to current drinkers (HR 1.14, 95% CI 1.00–1.30, p 0.049 after adjustment). Further analyses stratifying current drinkers by number of drinks per week did not significantly affect our results.

Supplemental analyses examining alcohol type (beer, wine, liquor) consumed in the past or present demonstrated no associations with incident AF after multivariable adjustment. Estimated cumulative alcohol consumption (in kilograms of alcohol) over study follow-up was not associated with an increased risk of AF either before (HR 1.00, 95% CI 0.99–1.00, p 0.081) or after adjustment (HR 1.00, 95% CI 0.99–1.00, p 0.724).

## Discussion

In this community-based, prospective study of past alcohol consumption and incident AF, a longer duration of alcohol abstinence among former drinkers was associated with a lower risk of developing AF, whereas previously consuming alcohol for a longer duration and consuming a greater quantity of alcohol were each associated with a higher risk of developing AF.

Episodic heavy alcohol consumption, or binge drinking, has long been posited as a trigger for AF [18], and in recent years, researchers have begun to investigate the effects of chronic alcohol consumption on AF risk [8]. Though several studies have found an association between both heavy [6, 7] and moderate alcohol consumption [8] and incident AF, not all studies have been positive. Indeed, to our knowledge, the only major US community-based cohort studies to have reported on the relationship between alcohol and incident AF are: the Framingham Heart Study, where two examinations found an association [5, 10] and another did not [19], and the Cardiovascular Health Study [20], which failed to demonstrate a statistically significant relationship between current alcohol consumption and risk of AF.

This is the first report regarding alcohol and AF from ARIC, where the nature of the questions enabled a focus on past alcohol consumption. In our study, former drinkers were more likely to have other cardiovascular risk factors and comorbidities, such as current or former smoking, CAD, HF, HTN, and DM. Yet, they actually demonstrated lower rates of AF, including when adjusted for these comorbidities. Indeed, previous research has failed to focus on the potentially clinically relevant issue regarding alcohol cessation and whether or how abstinence from alcohol might influence AF risk. Our findings indicate that the numbers of years of drinking and the amount of alcohol consumed may confer an increased risk of AF, even when abstinence is later practiced, suggesting that there may be chronic remodeling effects of alcohol that do not rely on alcohol as an acute trigger. Every decade of alcohol abstinence was associated with an approximate 20% decreased risk of incident AF, or one can consider the decrease in risk to be about 2% per year. Our recent study of alcohol consumption and incident AF in the Offspring and Original Framingham Study cohorts found that an estimated 24% of the association between alcohol and AF risk was explained by left atrial enlargement, offering a potential mechanism for alcohol’s arrhythmogenic effects on the myocardium[10]. Further research into which drinkers experience atrial remodeling and whether left atrial size decreases in those who quit drinking is critical. In addition, future investigations may help identify patients particularly prone to alcohol-related AF, and, when done, targeted counseling to those patients may be especially effective. For a disease that affects millions and is one of the most important causes of stroke [3], identifying modifiable risk factors is especially important.

Of interest, we found no relationship between either current drinking or cumulative alcohol exposure and incident AF. While the explanation for this remains unknown, underascertainment of prevalent AF as a cause must be considered. Specifically, prevalent AF was determined by baseline ECG only and therefore paroxysmal AF patients or formerly cardioverted persistent patients who happened to be in sinus rhythm during their baseline visit would have been misclassified. If those true (yet underascertained) prevalent AF patients had ceased alcohol intake because of their AF, this could lead to the false observation that current alcohol consumption failed to predict “incident” AF. Indeed, past consumption predicted incident AF in unadjusted analysis; loss of statistical significance in that relationship appeared to be primarily mediated by smoking. Importantly, this potential limitation of underascertainment would not result in any false positive results regarding the relationship between alcohol consumption and AF and specifically should not explain the results observed regarding differences *within* the past consumption group and AF.

Several limitations of our study must be acknowledged. First, we used self-report data as a measure of participants’ past and current drinking behavior. Though self-report has been previously validated as an accurate, reproducible method of assessing alcohol consumption over an extended period of time [21, 22], it is possible that this introduced some error into our data. However, given that participants were unaware of how this alcohol information would be used, it seems unlikely that recall bias was present. Given that diagnosis of AF relied on primarily hospital discharge records, as well as death certificates and study visit ECGs, under-ascertainment of incident AF is likely. It is important to emphasize that, in general, this would lead to inadequate power (and therefore may explain the negative results regarding current alcohol use and cumulative use) but would not be expected to result in false positives—in other words, we do not believe that any of these limitations would explain any false positive findings regarding the primary focus of this paper, alcohol abstinence duration and incident AF. In addition, given that data regarding paroxysmal versus persistent AF was not collected, we cannot comment on how alcohol might influence the risk of one type of AF versus another. A final limitation is our lack of data surrounding participants’ reasons for quitting drinking, as this may help explain the higher risk of AF among former drinkers. However, this again (if indeed the case) would not be sufficient to explain a lower risk of AF with a longer duration of abstinence.

## Conclusions

In this study of over 15,000 ARIC participants, we found that among former drinkers, the number of years of drinking and the amount of alcohol consumed each conferred an increased risk of AF. Finally, a longer duration of abstinence was associated with a decreased risk of AF, indicating that earlier modification of heavy alcohol use may have a greater influence on AF prevention.
