# Correction: Past alcohol consumption and incident atrial fibrillation: The Atherosclerosis Risk in Communities (ARIC) Study

**DOI:** 10.1371/journal.pone.0190329

**Published:** 2017-12-21

**Authors:** Shalini Dixit, Alvaro Alonso, Eric Vittinghoff, Elsayed Z. Soliman, Lin Y. Chen, Gregory M. Marcus

The fourth author’s name appears incorrectly. The correct name is: Elsayed Z. Soliman. The correct citation is: Dixit S, Alonso A, Vittinghoff E, Soliman EZ, Chen LY, Marcus GM (2017) Past alcohol consumption and incident atrial fibrillation: The Atherosclerosis Risk in Communities (ARIC) Study. PLoS ONE 12(10): e0185228. https://doi.org/10.1371/journal.pone.0185228
